# Steep HIV prevalence declines among young people in selected Zambian communities: population-based observations (1995–2003)

**DOI:** 10.1186/1471-2458-6-279

**Published:** 2006-11-10

**Authors:** Charles Michelo, Ingvild F Sandøy, Kumbutso Dzekedzeke, Seter Siziya, Knut Fylkesnes

**Affiliations:** 1 Department of Community Medicine, School of Medicine, University of Zambia, Lusaka, Zambia; 2 Centre for International Health, University of Bergen, 5021 Bergen, Norway; 3 Central Statistical Office, Lusaka, Zambia

## Abstract

**Background:**

Understanding the epidemiological HIV context is critical in building effective setting-specific preventive strategies. We examined HIV prevalence patterns in selected communities of men and women aged 15–59 years in Zambia.

**Methods:**

Population-based HIV surveys in 1995 (n = 3158), 1999 (n = 3731) and 2003 (n = 4751) were conducted in selected communities using probability proportional to size stratified random-cluster sampling. Multivariate logistic regression and trend analyses were stratified by residence, sex and age group. Absence, <30% in men and <15% in women in all rounds, was the most important cause of non-response. Saliva was used for HIV testing, and refusal was <10%.

**Results:**

Among rural groups aged 15–24 years, prevalence declined by 59.2% (15.7% to 6.4%, P < 0.001) in females and by 44.6% (5.6% to 3.1%, P < 0.001) in males. In age-group 15–49 years, declines were less than 25%. In the urban groups aged 15–24, prevalence declined by 47% (23.4% to 12.4%, P < 0.001) among females and 57.3% (7.5% to 3.2%, P = 0.001) among males but were 32% and 27% in men and women aged 15–49, respectively. Higher educated young people in 2003 had lower odds of infection than in 1995 in both urban [men: AOR 0.29(95%CI 0.14–0.60); women: AOR 0.38(95%CI 0.19–0.79)] and rural groups [men: AOR 0.16(95%CI 0.11–0.25), women: AOR 0.10(95%CI 0.01–7.34)]. Although higher mobility was associated with increased likelihood of infection in men overall, AOR, 1.71(95%CI 1.34–2.19), prevalence declined in mobile groups also (OR 0.52 95%CI 0.31–0.88). In parallel, urban young people with ≥11 school years were more likely to use condoms during the last casual sex (OR 2.96 95%CI 1.93–4.52) and report less number of casual sexual partners (AOR 0.33 95%CI 0.19–0.56) in the last twelve months than lower educated groups.

**Conclusion:**

Steep HIV prevalence declines in young people, suggesting continuing declining incidence, were masked by modest overall declines. The concentration of declines in higher educated groups suggests a plausible association with behavioural change.

## Background

HIV prevalence patterns and trends derived from available country data in sub-Saharan Africa, has been useful in improving the understanding of national estimates as well as proving strong basis for strengthening and or adjusting interventions [1-3]. The predominant epidemiological surveillance system for estimating HIV prevalence trends at country level in sub-Saharan Africa is antenatal clinic (ANC) based [4-7]. Despite this widespread use of ANC-based trends, validity and accuracy concerns as well as changing HIV dynamics in general populations as the epidemic matures, continue to pause interpretation challenges when these estimates are extrapolated to the general population[6,8-10]. However, population-based data on trends in HIV prevalence and related risk factors are still limited because only a few serial surveys have been conducted[2,10-13]. Furthermore, potential biases are also likely to hamper validity and reliability of population-based HIV surveys[14].

Zambia has established a comprehensive system for following the dynamics of the HIV epidemic which includes ANC-based surveillance and population-based surveys measuring infection and risk concomitantly over time[5,10,15]. In addition, a nationally representative population-based HIV prevalence survey has been conducted[16]. Evidence from ANC-based data shows declining prevalence trend in young pregnant women, a pattern that is masking differential geographical trends overall[5,17]. However, when population-based trends are compared with ANC-based estimates from the same community, declines are steeper in young people in the general population[10]. Furthermore, population-based data shows a significant shift towards reduced risk differentials amongst higher educated groups[5,10,11,15,18]. Therefore, there is need to understand and explain the existing HIV prevalence patterns in order to build an information base for prevention as well as monitoring and evaluation.

We investigated population-based HIV prevalence trends in the general population in Zambia from 1995 to 2003

## Methods

### Population and sampling procedures

The data is from three serial population-based HIV surveys conducted in 1995 (n = 3158), 1998 (n = 3757) and 2003 (n = 4775) in selected rural (Kapiri Mposhi) and urban (Chelstone, Lusaka) areas in Zambia using stratified random-cluster sampling method. The detailed methods of the surveys conducted in 1995–6 and 1998–9 have been reported elsewhere[10,17]. The sampling frame consisted of 24 Standard Enumeration Areas (SEAs) with 2786 households in Chelstone and 26 SEAs (5225 households) in Kapiri Mposhi. The SEA defined the primary sampling unit (cluster) of the study. Using probability proportional to size, 10 SEAs in Chelstone and 5 SEAs in rural Kapiri Mposhi were initially (1995) selected for the baseline survey. The numbers of SEAs in rural Kapiri Mposhi were increased to 10 in 1999 and 2003. All household members aged 15–59 in the selected clusters were listed and invited to participate in the study.

### Data Collection

In the sampled clusters (SEAs), a personal structured interview was carried out with all eligible and willing household members in order to collect information on socio-demographic and behavioural characteristics. Information on people who were not found in the subsequent survey was collected. At the end of the interview the participants were asked to provide a saliva sample for HIV testing.

### Laboratory Investigation

A standard algorithm that uses two-test strategy was employed in all the survey rounds. In the 1995 survey, all saliva samples were tested using Gacelisa HIV 1 & 2 (Welcome Diagnostics, Dartford, Kent, U.K.) and in addition, 450 randomly selected samples were tested using Bionor HIV-1&2 (BIONOR AS) magnetic particle assay following modifications for saliva. The two test kits showed a 99.8% agreement. The accuracy of Gacelisa was validated based on paired saliva and serum samples collected from 494 antenatal clinic attendees, and both sensitivity and specificity were 100%[19]. In addition, all respondents who wanted to know their results had their serum tested also in addition to saliva in accordance with the requirement in this country. This was useful for both as a validation strategy as well as quality assurance strategy[11,19]. In cases of discordance, the test result from serum was considered final. In the 1999 and 2003 follow-up surveys, samples were tested using Bionor HIV 1 & 2 and Gacelisa was only used in prescribed circumstances as a second test. In all the survey rounds, 10% of negative and 10% of positive samples were periodically sampled and re-tested for exactness with initial results and a different and senior person from a different section of the reference laboratory did this. Furthermore, in 2003, a second saliva test was done all the saliva specimens that tested positive but had no corresponding serum results, and were only considered positive if this second test was positive also. In case of discordance, a third test was done to decide the result. Once collected, these specimens were stored in a central place and then transported once a week for testing at a national reference laboratory (University Teaching Hospital, Lusaka). This laboratory which is run with the help of Japanese International Co-operation Agency (JICA), is the main laboratory for the country and maintains strict quality assurance schedule.

### Analysis

Intercooled Stata version 8 (College Station, Texas, USA) was used and the cluster effect accounted for in the analyses. Prevalence was standardised for age using the national census (2000) in order to control for changes in the age structure between surveys. The Mantel-Haenszel chi square test with continuity correction was used to test for linear trends. Multiple logistic regression analyses were used to assess and estimate the sex and residence specific changes in odds of infection between 1995 to 2003 per strata using 1995 as the reference time. A mobility index (high or low) was constructed based on frequency of travel out of usual residence and duration a respondent lived at current residence. People who travelled out of station frequently and had lived at current residence for less than or equal to two years were labelled to have a higher mobility index. This group of people was used as a proxy group for the respondents who were absent during the surveys. Conversely, people who travelled out of their station rarely and lived in the current residence for ≥6 years were coded to have a lower mobility index. The distribution of age as a continuous variable conformed to normality as assessed by probability plots. Interactions were looked for using the likelihood ratio test and when identified, the terms were computed to allow estimation of the statistical effect of one of the variables separately for each level of the effect-modifying variable. Model diagnostics were done using the maximum likelihood estimation (MLE) and the Hosmer-Lemeshow goodness-of-fit. The variables in the model were age (for 15–24 years group) or age group (for 15–49 years category) with marital status, religion, employment status, travel history, length of time lived in current residence, migration origin (from town or village), and birth history for women, stratified by sex and residence. Although analyses were done in ages 15–59 years, the term "overall" was reserved for estimates in age group 15–49 years only.

### Ethics

The survey protocol received clearance from the National AIDS Research Council and the University of Zambia Research Ethics Committee. In addition, participation in the population-based HIV survey was based on informed oral consent except in 2003 when written consent was obtained. Respondents were counselled and informed that the testing was purely for research purposes and was to be handled anonymously. However, respondents who were interested to know their status were offered voluntary counselling and testing (VCT) and a blood specimen was collected for serum-based HIV testing. Unlike in 1995 and 1999 surveys, when respondents were offered VCT either at the clinic and or at home respectively, in 2003 VCT was only provided at home in light of acceptability findings of an earlier study[20].

## Results

### Participation and distribution

Details of participation in the 1995–6 and 1998–9 population-based surveys have already been published and overall refusal rates were less than 10% in both surveys[10,17]. In 2003, 2705 rural residents (1301 men; 1404 females) and 4086 urban residents (1861 men; 2225 females) were listed. Non-participation was due to absence (19.7%), interview refusals (3.4%) or refusal to give a saliva sample for HIV testing (6.6%). Those absent (mostly either at school, in hospital or travelled out temporarily) were 25.5% (334) of the rural males, 11.8% (166) in rural females, 28.3% (527) among urban males and 14% (311) in urban females. Of the *de facto *eligible and successfully interviewed population of rural males, rural females, urban males and urban females, saliva refusal rates were 3.6%, 5.6%, 9.8% and 11.1% respectively. Only the respondents that had completed the interview, tested for HIV infection and aged 15–59 years (n = 4751, 56% urban and 44% rural) were included in the final analysis in 2003 (Table 1).

### Age-specific HIV infection trends

**Figure 1:**
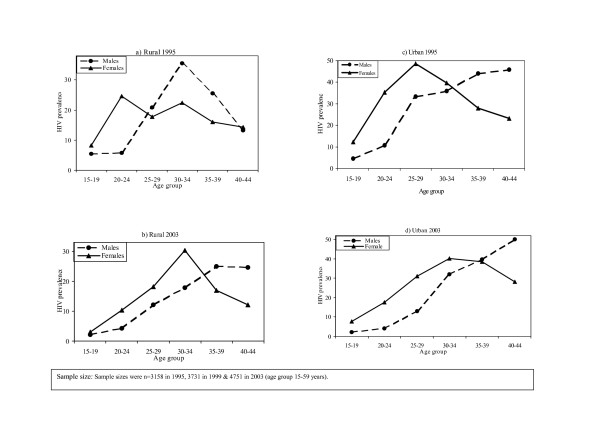
Age-specific HIV prevalence in rural and urban areas, illustrating cross-generational differences by sex: 1995 and 2003. This figure further illustrates the HIV prevalence declines by sex. Key to this are the increased age cross-over points, that's is, the point at which the HIV prevalence in young women which is usually higher than young men, is over taken by the prevalence in young men. Consequently we see a picture where after age 30s, HIV prevalence in men is higher than in women. Sexual relationships between these infected men and younger women under age 25 are probably the main force driving the HIV epidemic in sub-Saharan Africa.

Overall, HIV prevalence declined during this period irrespective of sex or residence (table 2). In urban groups aged 15–49 years, prevalence declined by 41% (21.4%, 18.3% to 12.6%; P < 0.001) in males and by 27% (29.6%, 27.0% to 21.7%; P < 0.001) in females. In the rural area, the declines were not statistically significant neither in males (15.3%, 13.3% to 13.0%; P = 0.314) nor females (17.4%, 14.4% to 14.1%; P = 0.118). In young people aged 15–24 years, prevalence declined by 44% (5.7%, 7.5% to 3.2%; P = 0.143) among males and by 58% (16.1%, 10.3% to 6.8%; P < 0.001) in females in the rural area. Similarly, it declined by 54% (6.9%, 7.4% to 3.2%; P = 0.005) among urban males and by 44% (22.5%, 18.3% to 12.5%; P < 0.001) in urban females. The age-specific HIV infection patterns showed a diverse picture. In age 40–49 and 50–59 years, prevalence increased in rural men but was stable in urban males. In rural females, prevalence declined in age groups 15–19 and 20–24 years and stabilised in groups aged 25 and above. However, in urban females, prevalence declined in groups under 30 years and stabilised in the 30–39 years old groups. Figure 1 illustrates this differential age-specific HIV prevalence declines further and worthy noting is the parallel decreasing prevalence by sex in young people by 2003 (figure 1).

### HIV infection trends by other socio-demographic characteristics

#### Level of education

In the urban group, aged 15–24 years with ≥10 school years, prevalence declined in both males (9.3%, 6.5% to 2.1%; P < 0.001; AOR 0.29 95%CI 0.14–0.60) and females (22.5%, 13.4% to 8.7%; P < 0.001; AOR 0.38 95%CI 0.19–0.79), table 3. Overall, the proportion with ≥10 school years (secondary education) was 7%, 6.5% and 11% in the rural area and 32%, 43% and 55% in the urban area in 1995, 1999 and 2003 respectively. The effect of education was significantly different between age groups (interaction P = 0.021 in 1995, P < 0.001 in 1999 & 2003). In the rural and urban groups aged 15–49 years, higher educated groups had lower odds of infection by 2003 in both sexes. No statistically significant trends were observed in neither young nor older respondents with 0–7 years of schooling (see table 4).

#### Marital status

In age 15–24, although married men had higher likelihood of infection than single respondents in both rural (AOR 2.31 95% CI 1.16–4.59) and urban areas (AOR 2.82 95%CI 2.1–3.83) in 1995, prevalence declined significantly in both rural (12.5, 11.3 to 5%) and urban (22.2, 14.3 to 8.3%) areas although the distribution by marital status did not change over the survey periods. In married females, prevalence declined also in both the rural (20.5, 10.0 to 7.6%) and urban areas (38.9, 26.0 to 22.5%). Similarly, prevalence declined significantly in urban married respondents aged 15–49 years (men, P = 0.035; women, P = 0.002), see Table 4.

#### Employment

Overall, men were more likely to be employed in both rural and urban areas. Although, employment was associated with increased likelihood of HIV infection in 2003 among rural males overall, AOR 1.67(95%CI 1.14–2.45), prevalence decline significantly irrespective of employment status in both sexes in the urban areas. In age 15–24 years, prevalence declined in all employment categories in both the rural and urban areas.

#### Mobility factors

In the age group 15–24 years, there were no significant differences in HIV prevalence patterns by mobility status. However, in age 15–49, the likelihood of infection among the more mobile groups tended to be higher than among the less mobile groups in men, AOR 1.45 (95%CI 0.98–2.18) in 1995, 1.75 (95%CI 1.17–2.6) in 1999 and 1.84 (95%CI 1.31–2.58) in 2003. Over the period, and among groups with higher mobility index, there was significant prevalence decline in the urban areas, but was non-significant in the rural area (men: 19.1%, 12.6% to 16.3%; P = 0.255; women: 20.0%, 15.9% to 16.4%, P = 0.289), see table 5. In respondents with ≥11 school years, the likelihood of infection was higher among mobile men in the urban area groups in 1995 (AOR 1.29 95%CI 0.53–3.16), 1999 (AOR 2.38 95%CI 1.1–5.27) and in 2003 (AOR 3.54 95%CI 1.41–8.86) than less mobile respondents. However, there were parallel prevalence declines in both the mobile (34%, 26.5% 14.5%, P < 0.001) and the less mobile group.

#### Fertility

The likelihood of infection in females aged 15–24 years who had given birth ever was higher than those who had never given birth in both rural (AOR 1.44 95%CI 0.56–3.7) and urban areas (OR 2.61 95%CI 1.71–3.99) in 1995. However, prevalence declined in both the group ever given birth (rural, P = 0.005; urban, P = 0.002) and the rural respondents who had never given birth (P = 0.001).

### Parallel behaviour changes in higher educated young people

Table 6 illustrates some of the corroborative sexual behaviour parallel changes observed in higher educated urban young people where there was declining HIV prevalence. The reported use of a condom during the last casual sex among young people with ≥11 school years increased from 42.7% to 67.3%, adjusted OR 2.96 95%CI 1.93–4.52 between 1995 and 2003 in comparison with groups with 0–7 school years. Similarly, higher educated young reported less number of casual sexual partners in the last twelve months than groups with 0–7 school years in 2003, AOR 0.33 95%CI 0.19–0.56. The observed changes in the rural area however, were less prominent although present.

## Discussion

A steep HIV prevalence decline was observed in young men and women aged 15–24 years in the studied populations. Prevalence declined also in urban men and women aged 25–29 years, whereas it was mostly stable in older age groups. In age group 15–49 years, a marked prevalence decline appeared in urban men and women, but the rural decline was modest and not significant. HIV-related mortality is likely to have contributed substantially to the overall decline. A 3-years cohort study based on the 1995 survey found HIV-related mortality to be generally low among young people, except in urban young women where HIV was found to have already affected mortality substantially (unpublished). In the absence of incidence data which was not possible to collect in this study due to ethical limitations, the observed prevalence declines in young people can be a proxy of change in incidence, and selective mortality seemed only to represent a small proportion of the steep decline in young urban women[21]. Similar patterns of declines have been reported in the region recently[1,2,5,13,22]. Furthermore, the declines were strongest in groups with secondary school education or more and were associated with sexual behavioural change.

It is possible that differential infection patterns among non-participants over time could have biased our estimates, but the magnitude and direction of this effect can only to some extent be assessed[2]. The non-participation due to refusal to provide saliva remained low in all survey rounds, and significantly below refusal levels as experienced in the Demography and Health Survey in Zambia 2001/2002 when using blood as the basis for HIV testing [16]. Absence was the most significant cause of non-participation, particularly in men. We think that the group of participants reporting to be highly mobile could be used as sentinel of HIV infection for men who were absent. Mobility and migration have been suggested to be factors fueling HIV transmission, but there are relatively few studies documenting it[23,24]. Mobile groups in this study had significantly higher likelihood of infection in men overall, but the risk difference was marginal in young men. However, we observed that even in these mobile groups there were significant prevalence declines in urban men and less prominent in the rural men. In younger people, we are persuaded to believe that if this bias was present, its effect was very minimal because the difference in odds of infection by mobility was non-significant and the major reason for absence was being away at school. Rather, it is likely that the prevalence among young people could even have been over-estimated due to the fact that those not found because of school attendance are less likely to be infected[25]. Notwithstanding the presence of selection biases due to non-response, they are unlikely to be an important factor explaining the sharp HIV prevalence declines among young people. We also note that where significant trends have been observed, all of the trends have been declining, irrespective of the factor being considered. This implies that there is no evidence of any interaction among the factors considered in this study.

External validity is a critical challenge when data from selected communities are extrapolated to the whole population. We found evidence supporting the interpretation that the HIV declines observed in these selected communities approximate well with those of the general population. Firstly, the prevalence levels of selected urban and rural communities matched with respective national estimates, and this was one of the criteria for selecting them[17]. Secondly, national ANC-based estimates show declines among young women[5]. Thirdly, we have previously reported that ANC-based trends under-estimate declines in the general population[10]. The main explanation for this reduced representativeness of ANC-based data was substantial delayed age at first birth among women in the general population.

HIV prevalence is a reflection of accumulations of infections over time, but during this same period, the population might change considerably[2]. Trend therefore reflects a time averaged dynamic balance between incidence, migration and mortality. The observed decline in prevalence could have been influenced by any or a combination of these factors. However, we realise that the changes were marked in young people where mortality is low and where there was no difference in odds of infection observed between in and out-migrants as observed elsewhere in the region[23]. Furthermore, the decline observed in higher educated young people who have adopted safer sexual practices is plausible because they have grown up during a time when prevention messages on HIV transmission were readily available, and this influenced their sexual behaviour[11]. In addition, we observe that if this trend continues, it has great potential to dictate further declines among educated people. HIV-related mortality's contribution to overall prevalence declines might reflect the impact of differential HIV-related mortality by age groups[2,26]. Long survival was unlikely to have any impact because access to anti-retroviral therapy was limited in this population before 2004.

Men acquired infections later than females and reached peak levels after age 30 years, and even among men aged 50–59 years prevalence levels remained very high. In this region, a gender-generational-power imbalance exists between casual and regular sexual partners. Men are usually older than their female partners and this age difference has been found to be "the major behavioural determinant of the more rapid rise in HIV prevalence in young women than in men"[27]. Most of the infections in young women are from older men in whom there has been a power change through age rise related affluence[27,28]. In order to control the HIV epidemic, breaking this cycle should be the cardinal aim of prevention programmes.

There are still limited data from sub-Saharan Africa linking HIV prevalence to parallel changes in risk behaviours[29]. We collected behavioural data and HIV status concomitantly and work on detailed parallel sexual behaviour patterns and HIV trends are the focus of a separate paper (awaiting publication). We found reductions in high risk behaviours among young people and that these were particularly in higher educated and urban groups. These findings confirm the assumption that effects of educational attainment on risk of HIV infection is likely to be exerted through mediator factors such as more consistent condom use, lower likelihood of sexually transmitted infections and less number of sexual partners[11,25,30].

## Conclusion

We conclude that the observed changes among young people suggest that HIV prevalence is really declining in these communities. This is unlikely to be due to either measurement bias or the natural course of the HIV epidemic given low mortality rates in this group, rather might be linked with prevention efforts[7]. The prevention programmes initiated in Zambia, mainly since the early 1990s, were complex and used multiple approaches largely focussing on behaviour change, but were limited in parallel poverty reduction strategies. In these communities where HIV transmission is largely heterosexual, a logical explanation that we derive from the proximate-determinant concept is that fear of HIV-related mortality combined with responses to prevention programmes may have influenced changes in behaviour thereby resulting in declines in HIV prevalence[30]. Key to this framework is the specification of a set of variables designated as "proximate determinants" (in this study it is condom use and number of partners) through which underlying factors operate to influence HIV prevalence. This interpretation is further supported by the fact that, over this period, transmission of HIV infection declined dramatically among higher educated people leading to a shift in the burden of infection from higher to lower educated groups. The changes observed among higher educated young people suggest that the availability of HIV preventive information was useful in forming their sexual behaviour, as they became sexually active after this critical information became well known[11]. An optimistic scenario is that these changes in higher educated groups will diffuse into those with lower education with time[31]. However, there is a negative association between educational attainment and poverty, manifested in this study by unchanging risk in lower educated and predominantly rural groups [32]. The link between HIV transmission and poverty might make such diffusion difficult. This suggests that prevention messages, though given, remain "irrelevant and inoperable" to many population groups whose poor economic and social conditions create an environment that might promote risky behaviours. Poverty reduction programmes, including strategies to increase educational attainment, are therefore to be seen as necessary components of effective HIV prevention efforts [32-34]. Furthermore, the observed prevalence decline is a sign that prevention works, and it should continue to be given "highest priority" among all other strategies including treatment efforts.

## Competing interests

The author(s) declare that they have no competing interests in whatever form.

## Authors' contributions

CM participated in the conception of the study, carried out the statistical analysis and drafted the manuscript. IFS reviewed all the drafts for intellectual content and participated in the interpretation of the findings. KD participated in the acquisition of data and review of the manuscript. SS participated in the critical review of the manuscript and was the lead person in the statistical handling and interpretation. KF conceived of the study, and participated in its design and co-ordination and helped to draft the manuscript. All authors read and approved the final manuscript.

## Pre-publication history

The pre-publication history for this paper can be accessed here:



## Figures and Tables

**Table 1:** Overall (Age group, ≥15 years) participation and response rates (percentages) of the population-based surveys conducted between 1995–2003 in selected rural and urban areas of Zambia

		**1995**	**1999**	**2003**
**Listed**		5847	6235	6791
**Absent**		927 (15.9%)	1763 (28.3%)	1346 (19.8%)
***De facto *eligible (found)**		4920	4472	5445
**Refused interview**		108 (2.2%)	53 (1.2%)	185 (3.4%)
**Interviewed**		4812 (97.8%)	4419 (98.4)	5260 (96.6%)
**Saliva test**	Accepted	4499 (93.5%)	4021 (91%)	4913 (93.4%)
	Refused	281(5.8%)	397 (9%)	347 (6.6%
	Lost	32 (0.7%)	--	--
	Damaged/Mislabelled	162	264	162
**Sample **(Interviewed & tested, Less damaged/mislabelled) §		4337 (88.2%)	3757 (84%)	4751 (87.3%)
**Final sample age 15–59 years**		3158	3731	4751

**Response (%) **(Listed-[absent+refusals]/listed*100)		77.4%	64.5%	72.3%

Notes: 1. §Percentages in the final sample analysed represents proportion of participation out of the de facto eligible population. This includes respondents aged ≥60 years in 1995 & 1999. 2. Information by sex was incomplete in the file linking the listed and interviewed respondents.

**Table 2:** Population based age-specific prevalence differential trends of HIV infection by sex in Chelstone, Lusaka and Kapiri Mposhi, Zambia: 1995–2003

Sex	Age group (years)	Age-specific Prevalence, % (total number of respondents, n)									
		
		Rural					Urban				
		
		1995	1999	2003	χ^2 ^for trend (P value)	Age-adjusted OR^ψ ^for 2003 (Ref., 1995)	1995	1999	2003	χ^2 ^for trend (P value)	Age-adjusted OR^ψ ^for 2003 (Ref., 1995)
Males	15–19	5.5 (73)	5.4(129)	2.1(146)	0.161		4.5(266)	6.3(224)	2.2(314)	0.144	
	20–24	5.8(103)	9.4(139)	4.3(163)	0.468		10.7(168)	8.6(208)	4.2(309)	**0.006**	
	25–29	20.9(67)	13.2(114)	12.2(147)	0.127		33.3(114)	22.0(109)	13.1(160)	0.001**	
	30–39	31.9(91)	23.1(169)	20.8(235)	**0.048**		40.0(150)	39.2(120)	35.4(181)	0.378	
	40–49	12.3(65)	12.9(101)	22.9(131)	**0.036**		41.5(106)	40.2(82)	33.3(78)	0.276	
	50–59	14.6(48)	9.3(92)	17.9(89)	0.416		26.5(34)	13.5(37)	25.8(66)	0.847	
											
	15–24										
	Crude	5.7(176)	7.5(268)	3.2(309)	0.143	0.56(0.25–1.26)	6.9(434)	7.4(432)	3.2(623)	**0.005**	**0.40(0.25–0.66)**
	Adjusted†	5.6	7.2	3.1	0.141		7.5	7.4	3.2	**0.001**	
	F:M ratio	2.8	1.46	2.06			3.12	2.47	3.87		
	Urban:Rural ratio	1.34	1.03	1.03			-----	-----	-----		
	15–49										
	Crude	15.3(399)	13.3(652)	13.0(822)	0.314	0.80(0.55–1.18)	21.4(804)	18.3(743)	12.6(1042)	0.000**	**0.58(0.38–0.87)**
	Adjusted†	15.4	12.8	11.6	0.072		24.8	18.3	16.9	**0.000**	
	F:M ratio	1.12	1.10	1.15			1.23	1.49	1.35		
	Urban:Rural ratio	1.61	1.43	1.46			-----	-----	-----		

Females	15–19	8.2(122)	5.9(217)	3.1(223)	**0.039**		12.3(391)	9.5(336)	7.7(431)	**0.026**	
	20–24	24.6(114)	15.9(163)	10.3(233)	**0.001**		35.4(311)	27.9(305)	17.6(409)	0.000**	
	25–29	17.7(79)	20.6(160)	18.2(187)	0.929		48.7(199)	41.2(221)	31.1(286)	0.000**	
	30–39	19.8(111)	18.4(233)	23.8%(256)	0.249		34.5(310)	39.3(244)	39.6(273)	0.202	
	40–49	17.8(73)	11.7(120)	14.7(156)	0.140		26.0(100)	22.3(112)	22.9(148)	0.614	
	50–59	15.4(52)	6.9(110)	5.3(94)	**0.043**		0(11)	25.0(12)	13.9(36)	0.444	
											
	15–24										
	Crude	16.1(236)	10.3(380)	6.8(456)	0.000**	**0.36(0.19–0.67)**	22.5(702)	18.3(641)	12.5(840)	0.000**	**0.43(0.33–0.57)**
	Adjusted†	15.7	10.5	6.4	0.000**		23.4	18.3	12.4	0.000**	
	Urban:Rural ratio	1.49	1.74	1.94			------	------	------		
	15–49										
	Crude	17.4(499)	14.4(893)	14.1(1055)	0.118	0.76(0.47–1.23)	29.6(1311)	27.0(1218)	21.7(1547)	0.000**	**0.68(0.57–0.81)**
	Adjusted†	17.3	14.1	13.4	0.056		30.6	27.3	22.8	0.000**	
	Urban:Rural ratio	1.78	1.94	1.70							

Notes **1**. † Denotes standardised prevalence using the Zambia National Census for the year 2000 as reference population **2. **OR^ψ ^stands for Odds ratio given with 95%Confidence Interval and adjusted with age as a continuous variable and confidence interval adjusted for clustering effect using Intercooled Stata version 8 with the Standard Enumeration Area (SEA) as clusters. Statistically significant OR, highlighted in bold **3. **Chi square for linear trend, Extended Mantel-Haenszel (1 degree of freedom) with continuity correction was used: **a) **Statistically significant trends are highlighted in bold b) ** denotes *P *< 0.001 **4. **Sample sizes were n = 3158 in 1995, 3731 in 1999 & 4751 in 2003 (age group 15–59 years).

**Table 3:** Trends in HIV prevalence by selected socio-demographic characteristics in rural and urban areas of Zambia, results of multivariate logistic regression and chi square tests for linear trend, 1995–2003: Age group15–24 years

			**Males**			**Females**		
			
			**Adjusted Odds Ratio(95%CI): reference time, 1995**		^§^**MH χ^2 ^for Linear trend, 1995–2003:**	**Adjusted Odds Ratio(95%CI): reference time, 1995**		^§^**MH χ^2 ^for Linear trend, 1995–2003:**
			1999	2003	P value	1999	2003	P value

**R**	**Education (~age)**‡							
		0–7 years	2.05(0.63–6.61)	0.69(0.17–2.80)	0.495	0.66(0.34–1.26)	**0.43(0.19–0.93)**	0.084
**U**		8–9 years	0.84(0.34–2.04)	0.40(0.09–1.78)	0.670	0.86(0.38–1.93)	0.25(0.05–1.26)	**0.002**
		≥10 years	1.26(0.11–13.8)	**0.16(0.11–0.25)**	0.113	0.29(0.08–11.1)	0.10(0.01–7.34)	**0.004**
**R**	**Marital status**	Single	1.57(0.57–4.36)	0.58(0.21–1.64)	0.383	1.45(0.29–7.24)	0.36(0.04–2.98)	**0.028**
		Married	0.79(0.22–2.88)	0.14(0.02–1.30)	0.187	**0.42(0.23–0.76)**	**0.29(0.13–0.68)**	0.001*
**A**	**Employed**	Yes	0.75(0.36–1.59)	0.38(0.13–1.09)	0.194	1.59(0.15–17.2)	0.43(0.04–5.01)	0.215
		No	2.30(0.77–6.90)	0.44(0.13–1.56)	0.338	0.65(0.37–1.13)	**0.33(0.14–0.78)**	0.001*
	**Travels**	Never	1.79(0.65–4.92)	0.68(0.31–1.48)	0.496	0.56(0.9–1.58)	**0.35(0.13–0.97)**	0.089
**L**		Sometimes	1.06(0.41–2.77)	**0.18(0.05–0.60)**	0.259	0.62(0.27–1.45)	**0.27(0.11–0.68)**	0.001*
		Often	1.21(0.49–2.99)	0.44(0.15–1.33)	0.396	0.47(0.16–1.39)	0.21(0.01–4.91)	0.113
	**Duration lived**	<1 year	#*	0.32(0.01–22.1)	0.649	0.33(0.08–1.39)	0.41(0.08–2.14)	0.087
	(In current residence)	1–5 yrs	1.77(0.53–5.92)	0.65(0.12–3.41)	0.565	0.85(0.39–1.88)	**0.34(0.12–0.97)**	0.058
		≥6 yrs	1.92(0.43–8.56)	0.28(0.03–2.31)	0.184	0.51(0.15–1.72)	**0.23(0.08–0.69)**	**0.003**
	**Migration**	Village	1.12(0.42–2.99)	1.07(0.30–3.79)	0.711	**0.44(0.21–0.90)**	**0.18(0.06–0.55)**	**0.002**
	(origin 5 years)	Town	0.59(0.03–9.98)	**0.01(0.00–0.13)**	0.456	1.46(0.32–6.52)	0.75(0.20–2.77)	0.494
	**Given birth**	Yes	--	--		**0.65(0.39–1.08)**	**0.42(19–0.89)**	**0.005**
	(ever): age, 15–24	No	--	--		**0.45(0.21–0.96)**	**0.09(0.02–0.51)**	**0.001**

**U**	**Education (~age)**‡							
		0–7 years	**6.69(2.47–18.1)**	1.45(0.22–9.40)	0.634	0.65(0.39–1.07)	0.79(0.43–1.43)	0.674
**R**		8–9 years	0.42(0.06–2.78)	0.57(0.22–1.49)	0.885	1.20(0.66–2.18)	0.59(0.33–1.07)	0.110
		≥10 years	0.94(0.45–1.95)	**0.29(0.14–0.60)**	0.001*	0.79(0.34–1.79)	**0.38(0.19–0.79)**	0.001*
**B**	**Marital status**	Single	1.29(0.66–2.53)	**0.56(0.32–0.95)**	**0.011**	1.09(0.68–1.74)	**0.57(0.36–0.88)**	**0.002**
		Married	1.03(0.69–1.55)	0.70(0.46–1.06)	0.306	**0.49(0.30–0.80)**	**0.38(0.25–0.58)**	**0.003**
**A**	**Employed**	Yes	1.89(0.35–10.2)	0.98(0.25–3.93)	0.454	1.02(0.39–2.66)	**0.41(0.19–0.86)**	0.232
		No	1.07(0.51–2.23)	**0.41(0.23–0.73)**	**0.003**	0.86(0.56–1.32)	**0.53(0.34–0.83)**	0.001*
**N**	**Travels**	Never	1.12(0.48–2.60)	0.79(0.34–1.87)	0.208	0.96(0.66–1.40)	**0.58(0.39–0.85)**	**0.025**
		Sometimes	1.45(0.34–6.19)	**0.16(0.04–0.71)**	**0.003**	0.86(0.51–1.45)	**0.46(0.26–0.81)**	0.001*
		Often	0.86(0.13–5.63)	0.99(0.19–5.21)	0.983	0.38(0.02–9.51)	0.32(0.06–1.84)	0.001*
	**Duration lived**	<1 year	4.67(0.93–23.2)	0.51(0.16–1.62)	0.571	0.87(0.44–1.74)	0.66(0.33–1.35)	0.638
	(In current residence)	1–5 yrs	0.63(0.23–1.72)	**0.33(0.16–0.69)**	**0.034**	**0.68(0.48–0.96)**	**0.42(0.26–0.67)**	0.001*
		≥6 yrs	1.60(0.22–11.5)	1.38(0.23–8.26)	0.353	1.26(0.46–3.40)	0.47(0.15–1.45)	0.001*
	**Migration**	Village	1.49(0.37–6.12)	0.46(0.07–2.98)	0.902	1.19(0.32–4.45)	1.09(0.24–4.99)	0.865
	(origin 5 years)	Town	1.08(0.31–3.74)	**0.38(0.18–0.79)**	0.068	0.71(0.48–1.08)	**0.47(0.30–0.74)**	0.001*
	**Given birth**	Yes	--	--		**0.65(0.51–0.86)**	**0.47(0.30–0.74)**	**0.002**
	(ever): age, 15–24	No	--	--		0.96(0.67–1.38)	**0.65(0.41–0.98)**	0.129

**Notes: 1. **^§^**MH **denotes Mantel-Haenszel chi square for linear trend, (1 degree of freedom) with continuity correction and statistically significant trends are highlighted in bold b) *signifies P < 0.001 **2. **Variables included in the model per strata were age, marital status, employment, religion, travel patterns, duration lived in current residence, migration origin in last five years and ever given birth for females **3. **^‡ ^The effect of education was significantly different between age groups (interaction P = 0.021 in 1995, P < 0.001 in 1999&2003), hence the "age*education" interaction term included in the models. **4. **Samples size in 1995, 1999 and 2003 and were 1548, 1721 and 2228 respectively **5. **#* No respondent had HIV in 1999 (n = 32)

**Table 4:** Trends in HIV prevalence by selected socio-demographic characteristics in rural and urban areas of Zambia, results of multivariate logistic regression and chi square tests for linear trend, 1995–2003: Age group 15–49 years

			**Males**			**Females**		
			
			**Adjusted Odds Ratio(95%CI): reference time, 1995**		^§^**MH χ^2 ^for Linear trend, 1995–2003:**	**Adjusted Odds Ratio(95%CI): reference time, 1995**		^§^**MH χ^2 ^for Linear trend, 1995–2003:**
			1999	2003	P value	1999	2003	P value

**R**	**Education (~age)**‡							
		0–7 years	0.93(0.62–1.34)	0.72(0.47–1.09)	0.227	1.07(0.57–2.01)	1.03(0.62–1.72)	0.838
**U**		8–9 years	0.74(0.29–1.88)	0.84(0.28–2.46)	0.731	0.52(0.24–1.13)	0.50(0.22–1.14)	0.104
		≥10 years	**0.38(0.15–0.98)**	**0.44(0.22–0.87)**	0.223	**0.33(0.17–0.65)**	**0.29(0.08–0.96)**	**0.043**
**R**	**Marital status**	Single	1.48(0.76–2.86)	0.50(0.22–1.12)	0.188	0.74(0.23–2.4)	0.31(0.06–1.49)	**0.021**
		Married	0.66(0.38–1.15)	**0.63(0.42–0.96)**	0.096	0.91(0.53–1.57)	0.82(0.49–1.37)	0.112
**A**	**Employed**	Yes	0.66(0.42–1.03	0.76(0.48–1.21	0.914	0.81(0.21–3.15)	0.73(0.22–2.42)	0.479
		No	1.03(0.57–1.84)	**0.55(0.34–0.89)**	**0.026**	0.82(0.48–1.38)	0.78(0.47–1.31)	**0.055**
	**Travels**	Never	0.99(0.52–1.91)	1.36(0.86–2.16)	0.180	0.93(0.54–1.60	1.09(0.70–1.71)	0.644
**L**		Sometimes	**0.66(0.44–0.98)**	**0.43(0.27–0.70)**	**0.019**	0.76(0.33–1.71)	0.59(0.31–1.10)	**0.024**
		Often	0.76(0.21–2.75)	0.62(0.19–1.94)	0.563	0.92(0.23–3.67)	0.44(0.07–2.89)	**0.051**
	**Duration lived**	<1 year	**0.25(.10–0.63)**	0.63(0.22–1.81	0.733	**0.57(0.33–0.99)**	0.79(0.35–1.82)	0.199
	(In current residence)	1–5 yrs	0.74(0.36–1.52)	0.57(0.26–1.28)	0.069	1.79(0.88–3.62)	1.41(0.74–2.69)	0.234
		≥6 yrs	1.24(0.76–2.03)	0.95(0.60–1.51)	0.937	**0.45(0.23–0.89)**	**0.48(0.26–0.88)**	**0.011**
	**Migration**	Village	0.72(0.43–1.22)	0.99(0.51–1.94)	0.922	1.12(0.58–2.16)	1.04(0.55–1.97)	0.840
	(origin 5 years)	Town	**0.48(0.22–0.98)**	**0.30(0.13–0.72)**	**0.041**	1.75(0.99–3.01)	1.31(0.75–2.32)	0.586
	**Given birth**	Yes	--	--		0.75(0.42–1.34)	0.58(0.30–1.11)	**0.019**
	(ever): age, 15–29	No	--	--		0.56(0.23–1.33)	**0.27(0.07–0.99)**	**0.012**

**U**	**Education (~age)**‡							
		0–7 years	1.66(0.80–3.42)	1.34(0.72–2.49)	0.398	0.83(0.59–1.16)	1.11(0.71–1.76)	0.498
**R**		8–9 years	0.42(0.14–1.26)	0.52(0.26–1.06)	0.305	1.10(0.76–1.61)	0.66(0.39–1.08)	0.179
		≥10 years	1.03(0.72–1.49)	**0.55(0.40–0.74)**	0.001*	0.91(0.61–1.34)	**0.53(0.39–0.72)**	0.001*
**B**	**Marital status**	Single	0.95(0.62–1.46)	**0.51(0.33–0.79)**	0.001*	1.05(0.69–1.58)	**0.49(0.35–0.69)**	0.001*
		Married	1.04(0.72–1.52)	**0.71(0.47–0.98)**	**0.035**	0.92(0.74–1.14)	**0.73(0.57–0.94)**	**0.002**
**A**	**Employed**	Yes	0.97(0.69–1.36)	**0.64(0.44–0.95)**	**0.001**	0.98(0.67–1.41)	**0.69(0.51–0.95)**	**0.018**
		No	0.99(0.60–1.64)	**0.52(0.37–0.74)**	0.001*	0.93(0.76–1.15)	**0.63(0.47–0.85)**	0.001*
**N**	**Travels**	Never	0.81(0.57–1.16)	0.57(0.32–1.02)	**0.001**	0.88(0.70–1.11)	**0.64(0.49–0.83)**	**0.003**
		Sometimes	1.14(0.86–1.49)	**0.52(0.34–0.81)**	0.001*	1.06(0.84–1.35)	**0.71(0.52–0.97)**	**0.008**
		Often	1.11(0.55–2.21)	0.80(0.38–1.70)	0.189	0.77(0.23–2.59)	**0.44(0.23–0.82)**	**0.002**
	**Duration lived**	<1 year	1.62(0.83–3.19)	**0.47(0.28–0.76)**	**0.052**	0.83(0.54–1.27)	**0.66(0.45–0.97)**	0.196
	(In current residence)	1–5 yrs	0.85(0.54–1.35)	0.71(0.47–1.09)	**0.007**	0.83(0.59–1.16)	**0.56(0.40–0.79)**	0.001*
		≥6 yrs	0.90(0.56–1.46)	**0.53(0.29–0.99)**	0.001*	1.31(0.95–1.81)	0.86(0.57–1.33)	0.129
	**Migration**	Village	1.01(0.39–2.59)	0.39(0.09–1.54)	0.314	0.98(0.42–2.32)	0.93(0.42–2.09)	0.651
	(origin 5 years)	Town	1.03(0.63–1.68)	**0.69(0.49–0.96)**	**0.001**	0.83(0.64–1.06)	**0.58(0.45–0.74)**	0.001*
	**Given birth**	Yes	--	--		**0.73(0.63–0.35)**	**0.51(0.38–0.67)**	0.001*
	(ever): age, 15–29	No	--	--		0.87(0.64–1.19)	**0.52(0.35–0.75)**	**0.042**

**Notes: 1. **^§^**MH **denotes Mantel-Haenszel chi square for linear trend, (1 degree of freedom) with continuity correction and statistically significant trends are highlighted in bold b) *signifies P < 0.001 **2. **Variables included in the model per strata were age group, marital status, employment, religion, travel patterns, duration lived in current residence, migration origin in last five years and ever given birth for females **3. **^‡ ^The effect of education was significantly different between age groups (interaction P = 0.021 in 1995, P < 0.001 in 1999&2003), hence the "age*education" interaction term included in the models. **4. **Samples size in 1995, 1999 and 2003 and were 3013, 3506 and 4466 respectively

**Table 5:** Population-based HIV prevalence trends of groups with high mobility index^¶ ^by sex in selected areas in Zambia, 1995–2003

**Sex**	**Residence**	**Year**	**HIV prevalence (n)**	**Age-adjusted Odds Ratio (OR)^ψ ^for likelihood of HIV infection in 1999 and 2003 (Reference, 1995)**	**χ^2 ^for linear trend (P value)**
Males	Rural	1995	19.1(94)	1.00	0.255
		1999	12.6(87)	0.59(0.26–1.33)	
		2003	16.3(104)	0.72(0.34–1.54)	
	Urban	1995	23.0(226)	1.00	**0.036**
		1999	23.4(107)	0.98(0.56–1.75)	
		2003	14.7(197)	**0.52(0.31–0.88)**	

Females	Rural	1995	20.0(65)	1.00	0.289
		1999	15.9(107)	0.74(0.33–1.66)	
		2003	16.4(128)	0.77(0.35–1.67)	
	Urban	1995	35.0(314)	1.00	**0.009**
		1999	28.1(139)	0.75(0.48–1.18)	
		2003	25.0(252)	**0.63(0.43–0.92)**	

Notes **1**. ^¶ ^High mobility index was a composite measure of groups who travelled out of station frequently for any reason and have lived at current residence for less or equal to two years. **2. **OR^ψ ^was adjusted for age and school years (as a continuous variable), employment status and marital status. Statistically significant OR highlighted in bold **3. **Extended Mantel-Haenszel Chi square for linear trend, (1 degree of freedom) with continuity correction was used; highlighted in bold is statistically significant P value **4. **Sample sizes were n = 585 in rural and 1235 in urban (age group 15–49 years).

**Table 6:** Likelihood of "condom use at last casual sex" and "having ≥2 sexual partners in last 12 months" by educational attainment among urban respondents aged 15–24 years in Zambia, results of multivariate logistic regression: 1995–2003

**Factor**	**Education**	**1995**		**1999**		**2003**	
		
		**Prevalence [%(n)]**	**Adjusted Odds Ratio(95%CI)**	**Prevalence [%(n)]**	**Adjusted Odds Ratio(95%CI)**	**Prevalence [%(n)]**	**Adjusted Odds Ratio(95%CI)**
**Condom use during the last casual sex**	0–7 years	25.5 (286)	1	26.2(126)	1	42.9(119)	1
	8–10 years	27.4 (402)	0.98(0.69–1.43)	51.2 (201)	2.82(1.73–4.60)	53.8 (184)	1.54 (0.96–2.46)
	≥11 years	42.7(218)	1.40(0.94–2.10)	68.9 (270)	5.62(3.44–9.20)	67.3 (437)	**2.96(1.93–4.52)**

**Proportion having ≥2 sexual partners, last 12 months**	0–7 years	16.2 (376)	1	11.0 (145)	1	20.8 (144)	1
	8–9 years	16.2 (469)	0.76(0.51–1.13)	18.4 (228)	1.48(0.78–2.82)	16.7 (204)	0.63(0.35–1.12)
	≥11 years	15.6 (270)	0.49(0.30–0.79)	17.2 (285)	1.06(0.55–2.82)	11.5 (442)	**0.33(0.19–0.56)**

**Notes: 1. **All confidence interval adjusted for clustering effect using Intercooled Stata version 8 with the Standard Enumeration Area (SEA) as clusters **3. **Variables included in the model were education, age, sex, marital status, employment and religion. **2. **Samples sizes (were drawn from groups ever had sex).
